# Interaction of Viral Capsid-Derived Virus-Like Particles (VLPs) with the Innate Immune System

**DOI:** 10.3390/vaccines6030037

**Published:** 2018-07-02

**Authors:** Mona O. Mohsen, Ariane C. Gomes, Monique Vogel, Martin F. Bachmann

**Affiliations:** 1 Jenner Institute, University of Oxford, Oxford OX3 7BN, UK; ariane.cruzgomes@kellogg.ox.ac.uk (A.C.G.); martin.bachmann@ndm.ox.ac.uk (M.F.B.); 2 Qatar Foundation, Doha, Qatar; 3 Inselspital, Universitatsklinik RIA, Immunologie, 3010 Bern, Switzerland; monique.vogel@dbmr.unibe.ch

**Keywords:** virus-like particle (VLP), innate immune system, adaptive immune system, Toll-like receptor (TLR)

## Abstract

Virus-like particles (VLPs) derived from viral nucleocapsids are an important class of nanoparticles. The structure, uniformity, stability, and function of these VLPs have attracted scientists in utilizing them as a unique tool in various applications in biomedical fields. Their interaction with the innate immune system is of major importance for the adaptive immune response they induce. The innate immune cells and molecules recognize and interact with VLPs on the basis of two major characteristics: size and surface geometry. This review discusses the interaction of viral capsid-derived VLPs with the innate immune system.

## 1. Virus-Like Particles (VLPs)—Brief Overview

Virus-like particles (VLPs) are supramolecular complexes formed by viral proteins that self-assemble into capsids resembling real viruses, albeit non-infectious due to the lack of viral genome packaged within the particle. Such non-infectious particles can be naturally formed during infection or genetically engineered and produced in large scales in laboratories. Most VLPs are characterized by stability, together with uniform and repetitive structure that allow diverse applications in the field of biomedical science. Since their early discovery five decades ago, VLPs led to enormous progress in the field of vaccine development with seven vaccines approved for humans and several others in pre-clinical phase or in clinical trials [1,2]. Most VLPs are formed by proteins naturally forming nucleocapsids and consist of several copies of monomers in quasi-equivalent conformations forming icosahedral or helical (rod-shaped) structures. The final shape of VLPs usually imitates the symmetry of the original parental virus but can vary according to the nucleic acid content or biophysical context during the assembly phase [3].

**Figure 1:**
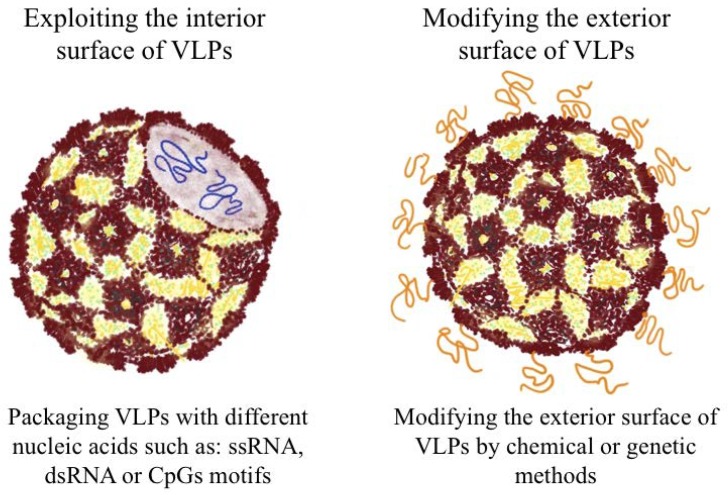
A schematic diagram of nucleocapsid-derived VLPs illustrating some modifications that can enhance their immunogenicity. (1) packaging of nucleic acids and (2) displaying heterologous proteins/epitopes or functional molecules on the outer surface.

The virus-like property of VLPs is a potent geometric pathogen-associated structural pattern (PASP) facilitating their engagement with innate and adaptive immune cells. The exterior and interior facets of VLPs can be functionalized and modified to enhance their immunogenicity and stability or to present heterologous antigens (Figure 1). The viral capsids have evolved to preferentially package their own viral genome. Nucleocapsid-derived VLPs retain this property of packaging nucleic acids, and since the viral genome is absent, VLPs will package nucleic acids from host cells during the expression process. Interestingly, these naturally packaged nucleic acids can be substituted by other anionic polymers to modulate the immune response by engagement of different pattern recognition receptors (PRRs) [4,5,6]. The outer surface of VLPs has also been functionalized with heterologous antigens by chemical crosslinking, genetic fusion or peptide splicing techniques aiming to enhance the immunogenicity of coupled antigens [6,7].

## 2. VLPs Interaction with the Innate Immune System

VLPs—especially those packaging nucleic acids—are capable of inducing a comparable humoral immune response to viruses. This is considered a major advantage of VLPs over attenuated virus-derived vaccines as it guarantees safety of the vaccines whilst retaining their immunogenicity [8]. The immune system in general recognizes and interacts with viral capsid-derived VLPs on the basis of two major characteristics, *size and surface geometry* [2] as discussed next.

### 2.1. Drainage of VLP into the Lymphatoid Organs

The lymphatic system regulates the balance of fluid in the body as well as the trafficking of immune cells and antigens to lymphoid organs, in particular lymph nodes (LNs) [9]. The efficient draining of antigens and particles from the periphery to the primary and secondary lymphoid organs guarantees the encounter of such particles with the relevant immune cells that will initiate the immune response. Thus, draining properties of antigens have an important impact on its overall immunogenicity [10,11].

The diameter of the blind-ended structure of the initial lymphatic vessels is about 10–60 μm. The vessels are lined up with single flattened endothelial cells forming valves that ensure the unidirectional movement of fluids through and along the lymphatic vessels [12]. Furthermore, the initial lymphatic vessels are highly permeable due to the presence of button-like junctions or gaps between the endothelial cells [13]. Particulate materials can directly diffuse through the 200 nm pores of the lymphatic vessels’ walls with an optimal size between 30–40 nm [14]. VLPs range between 20–200 nm in size, which allows them to freely drain into the lymphatic system.

The kinetics of free drainage of viral capsid-derived VLPs has been studied extensively and previous data has shown that VLPs with average size of 30 nm can be detected in mice footpads for at least 40 min post injection and at the draining LN 2 h post injection [14]. Recently, with the advancement of imaging techniques it has been shown that 30 nm VLPs can accumulate at the popliteal draining LN in less than 10 min post subcutaneous injection in murine footpads [15]. In addition to free lymphatic drainage, VLPs can also be actively transported via specialized cells such as skin-derived macrophages and DCs, mainly dermal DCs (dDCs) and Langerhans cells (LCs). These cells, especially upon activation, can efficiently squeeze through the button-like junctions of the endothelial cells lining lymphoid vessels and migrate through the lymph along a CCL19/CCL21 gradient towards the draining LN. Previous studies have shown that skin-derived DCs—CD11c^hi^CD40^hi^CD8^−^ and CD11c^int^CD40^hi^CD8^−^ DCs—can successfully uptake VLPs after intradermal injection. Both identified subsets of skin-derived DCs have also been shown to effectively cross-prime cytotoxic T-cells following VLPs uptake [16]. Even though, CD8^+^ DCs are usually much more potent in cross-presenting VLP-derived antigens [17].

In summary, the size of antigen influences the immunogenicity largely by two mechanisms: first, by allowing the appropriate draining from periphery to secondary lymphoid organs and second by facilitating the interaction with APCs due to their repetitive surface. VLPs size distribution falls within the aforementioned optimal range, which explains in part its remarkable immunogenicity.

### 2.2. Trafficking of VLPs Within Draining LN

**Figure 2:**
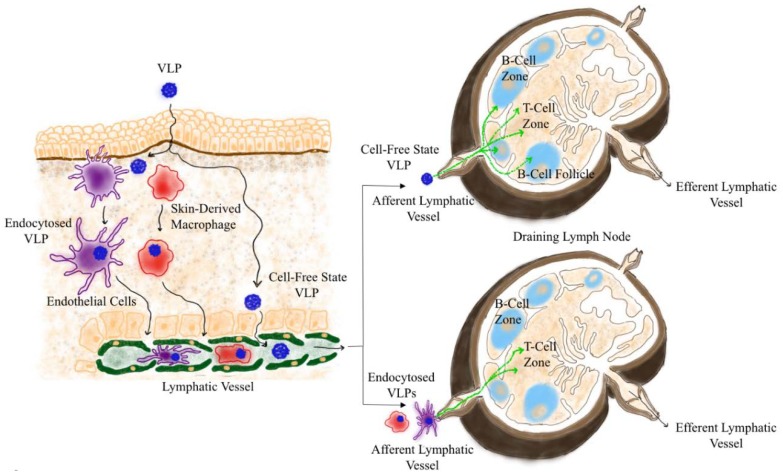
Distribution of VLPs in the draining LN. VLPs arrive in the LNs by natural drainage in a cell-free manner or transported by APCs. Upon reaching the sub-capsular sinus of the draining LN, VLPs will be phagocytized by different APCs and preferentially reach the B and/or T-cell zones. VLPs endocytosed in the periphery by APCs preferentially reach the T-cell zone in the draining LN for T-cell priming.

In general, particles ranging between 20–200 nm upon reaching the LN are distributed throughout the different areas of the draining LN. Previous studies have shown that VLPs are detected at the sub-capsular, medullar and cortical regions of LNs as fast as 2 h post subcutaneous injection. At a later stage, VLPs are also detected deeper in B-cell follicles bound to follicular dendritic cells (fDCs) (Figure 2). At 48 h post injection VLPs were distributed in the sub-capsular area, cortex, and para-cortex of the draining LN and some were inside B-cell follicles [14]. Upon arrival at the draining LN, several LN residing myeloid cells and B-cells will carry out the active transportation of free VLPs from the sub-capsular sinuses to B-cell follicles [18,19,20,21]. The pattern of distribution of VLPs allows access and interaction of particles with different cell types in the secondary lymphoid organs, which contributes to the immune response generated against those particles.

The predominant subsets of APCs actively up taking viral capsid-derived VLPs in the popliteal LN post subcutaneous injection have been recently classified. These cells include the sub-capsular sinus (SCS) macrophages CD11b^+^F4/80^+^, different subsets of conventional dendritic cells (cDCs) including CD8^+^CD11c^+^, CD8^−^CD11b^+^, and CD8^+^CD11c^+^. B-cells characterized by CD45R/B220^+^ were less efficient in up taking VLPs [15] but are likely responsible for their transport to fDCs in B-cell follicles [21]. Medullary DCs within lymphoid organs would also participate in the transportation of antigens by binding them to their surface receptors such as SIGN-R1 (CD209b) [20]. Other studies have shown that SIGN-R1 and DC-specific ICAM3-grabbing non-integrin receptors (DC-SIGN) have also a role in antigen transportation and presentation. In addition, DCs can also capture VLPs and present VLP-derived peptides in the paracortical T-cell zone resulting in the activation of T and B-cells in the extra-follicular area [22]. Initial studies have shown that skin-derived DCs which have encountered antigens in the skin preferentially colonize specific areas within the draining LN. dDCs are known to migrate to the paracortex underneath B-cell zone, whereas LCs migrate and colonize the inner-paracortex [23]. Therefore, actively transported VLPs by skin-derived DCs either dDCs or LCs would follow similar migration pattern to T-cell zone and thereby are considered to be key initiators of T-cell immunity. A study suggested that skin-derived DCs act as initial transporters of herpes simplex virus (HSV) to non-migratory LN resident CD8^+^ DCs for effective CTL priming [24].

As described above, the draining and trafficking of VLPs within the immune system and subsequent interaction with immune cells such as DCs, macrophages, and B cells allow the initiation of both humoral and cellular immune responses. Details of the immune response to VLPs used as vaccines will be discussed in the following sections.

### 2.3. VLPs and Innate Humoral Immune Response

**Figure 3:**
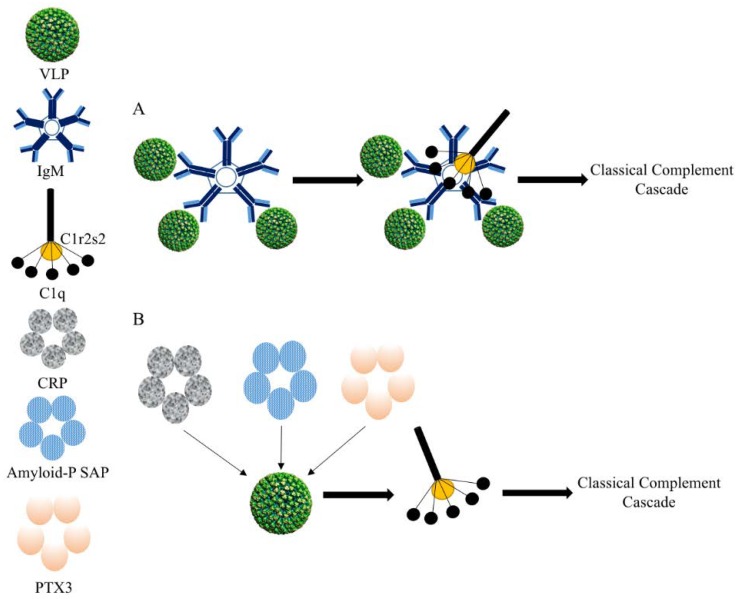
VLPs and innate humoral immune response. The repetitive surface of viral capsid-derived VLPs facilitates their interaction with molecules and components of the innate humoral immune system. (**A**) IgM binds the surface of VLPs via low affinity/high avidity interaction. This binding activates C1q molecules resulting in the initiation of the classical complement cascade. (**B**) More speculative, pentraxin protein family members including CRP, amyloid P SAP and PTX3 may also recognize and bind the repetitive surface structure of VLPs resulting in the activation of C1q molecule and the initiation of the classical complement cascade.

The highly repetitive surface structure of viral capsid-derived VLPs facilitates their interaction with components of the innate humoral immune system that in turn mediates opsonization and phagocytosis by APCs (Figure 3). The natural pentameric IgM antibody can efficiently bind to VLPs via low affinity/high avidity interactions [2,21]. It has been shown that even if the interaction between a single viral coat sub-unit with a single recognizing element on the multimeric molecule is weak, the overall avidity of the interactions of the antibodies with the entire VLP would be considerably stronger thanks to the repetitive surface of the particles [21]. The pentameric IgM alone is not an efficient direct activator of the opsonization mechanism. However, IgM contributes to the activation of C1q molecule upon binding. Activation of C1q consequently leads to the activation of the classical complement cascade initiated by the formation of C1-complex consisting of C1q and serine proteases C1r and C1s molecules. C1q can also bind directly to the surface of VLPs causing a conformational change in C1q molecule leading to the activation of classical complement cascade. In our hands, however, both deficiency in IgM as well as C1q led to a complete failure of VLP-deposition on fDCs [21]. Such findings indicate that natural IgM binds to VLPs followed by recruitment of C1q. Interestingly, complement may not always enhance activation of innate cells. Papaya Mosaic Virus VLPs for example induce marked production of IFN-α in pDCs and complement inhibits rather than promotes this process [25].

The pentraxin protein family including the short pentraxin C-reactive protein (CRP), serum amyloid P (SAP) and long pentraxin (PTX3) may also recognize the repetitive structure of VLPs due to high avidity interactions. SAP, for example, has been shown to bind to influenza A viral particles [26]. This recognition may also result in high affinity binding to C1q molecule and effective initiation of the classical complement cascade [2]. More research, however, is necessary to study the interaction of the innate humoral immune system and VLPs.

The hallmark of the immune response induced by VLPs is the induction of high levels of long lasting humoral responses. In contrast to soluble antigens, the highly organized and repetitive structure of VLPs promotes crosslinking of BCRs that surpasses the activation threshold and bypasses the initial need of T-cell help. As a consequence, any antigen exposed on the surface of VLPs in this same organized and repetitive array will benefit from this viral fingerprint and induce high levels of humoral responses.

Another important feature of the humoral response elicited by VLPs is the promotion of isotype switching dependent on direct TLR7 or TLR9 signaling on B-cells [27,28]. Upon BCR-mediated endocytosis of VLPs, the nucleic acid that is often packaged within VLPs reaches the endocytic compartment where it can interact and activate endosomal TLRs, the downstream response leads to isotype switching of antibodies to IgG2 in mice and IgG1 in humans, the isotypes with higher effector function.

Furthermore, intranasal administration of VLPs in murine models resulted in strong B-cell responses and germinal center formation in the spleen. Roaming B-cells in the lungs have been shown to bind VLPs through their BCR and shuttle them to B-cell follicles within the spleen [29].

### 2.4. Efficient Presentation of VLPs by Both MHC Pathways

Following the uptake by professional APCs, processed VLPs will be subsequently presented on MHC molecules. In general, exogenous antigens (endocytosed or phagocytosed) are presented on MHC-II molecules to trigger the activation of T_h_ CD4^+^ cells following endosomal processing [30]. Endogenous antigens on the other hand are loaded on MHC-I molecules for CD8^+^ CTLs priming. The classical source of MHC-I antigens is newly synthesized proteins which are misfolded and degraded in the proteasomes. Nevertheless, it has been found that both presentation pathways are not strictly separated [31] as exogenous antigens can also reach MHC-I presentation pathway in a process called cross-presentation [32,33,34]. Cross-presentation can be TAP dependent or independent. In TAP dependent pathways the antigen will be taken by endosomes and leak to the cytosol afterwards (endosome to cytosol pathway). In TAP independent pathway, peptide load MHC-I in the endosomes directly as an exchange process under acidic conditions, such a process may be referred to as direct endosomal loading pathway [34,35,36]. The ability of exogenous VLPs to be presented on MHC-II molecules to generate protective IgG antibody titers via activating T_h_ cells has been previously documented to be highly efficient and biologically relevant. Cross-presentation on MHC-I molecules has also been studied extensively and has been documented for tumor cells, necrotic cells, apoptotic cells, viruses, and VLPs [34,37,38,39]. The efficiency of VLPs presentation via MHC-I vs. MHC-II molecules in vivo was quantified [34]. This was done by using immunodominant MHC-I or MHC-II antigens derived from lymphocytic choriomeningitis virus (LCMV) linked to VLPs. The results indicated that cross presentation on MHC-I molecules for VLPs containing antigens was only 1 to 10-fold less efficient than classical presentation on MHC-II molecules. This finding indicates that VLPs as exogenous particles can be loaded efficiently onto both MHC molecules [34]. It has been reported that exogenous antigens can reach MHC-I molecules in CD8^+^ DCs only while CD8^−^ DCs present them on MHC-II molecules [40].

However, this dichotomy hypothesis was not supported in other studies which have shown that both CD8^+^ and CD8^−^ DCs can efficiently present VLP-derived antigens on MHC-II molecules, however only CD8^+^ DCs were able to cross-present VLPs derived peptides. This implies that VLPs presentation on MHC-II molecules to CD4^+^ T-cells is not restricted to a specific DCs subset but cross-presentation to CD8^+^ T-cells is usually restricted to a CD8^+^ DCs subset [17].

### 2.5. Packaging VLPs with Innate Immune-Modulators

Many VLPs, including Norovirus, Papaya Mosaic Virus, Human Papilloma Virus and Qβ-derived VLPs activate innate cells through various means [25,36,41]. An important reason for the ability of RNA virus nucelocapsid derived VLPs to activate DCs and B-cells is the fact that they package RNA. Even though these VLPs lack infectious genome, they usually package nucleic acids (RNA) during the assembly process in host cells. Moreover, VLPs can be efficiently packaged in vitro with a range of charged polyanionic sequences such as RNA with specific secondary modification or CpGs, for instance. Nucleic acids are recognized and can activate PRRs, which modulates and alters the adaptive immune response [42]. VLPs are reportedly inefficient at inducing strong antigen-specific cytotoxic response, however the addition of other certain classes of PRR ligands such as non-methylated CpGs can efficiently activate TLR9 leading to robust T_h_1 and CTL response [43]. Non-methylated CpGs are classified into three different categories according to their structure and the immune response they induce. In general, CpGs of all three classes can activate inflammatory cytokines as well as type-I IFN at different levels upon stimulation of TLR9 in plasmacytoid dendritic cells (pDCs) [44]. Class A CpGs are characterized by the presence of phosophodiester poly G sequence at the 5′ and 3′ ends as well as an internal palindrome sequence in the central portion. Class A CpGs are capable of producing large amounts of type-I IFN in particular by pDCs [45]. In contrast, class B CpGs characterized by a full phosphorothioate backbone can induce type-I IFN production to a lesser extent than class A. However, class B CpGs is capable of stimulating pro-inflammatory cytokines, such as IL-12 [46]. The homologous TLR7/8 expressed within the endosomal compartments of APCs can recognize guanine nucleotide-homologues such as loxoribine, R848, imidazouinoline components, and uridine/guanosine rich ssRNA [47]. Free RNA is usually subjected to degradation by the RNase enzyme in the extracellular spaces. Accordingly, the possibility that free RNA will reach the endosomal compartments of APCs to interact with and activate endosomal TLRs is quite low. Therefore, packaging ssRNA into VLPs protects it from extracellular RNase and results in efficient activation of TLR7/8. TLR3 is mainly expressed in cDCs but not pDCs and recognizes dsRNA. The synthetic RNA sequence poly I:C, leads to a type-I IFN response and production of proinflammatory cytokines [48]. It has been reported that VLPs formulated with Poly I:C can lead to an improved CTL response [49].

**Figure 4:**
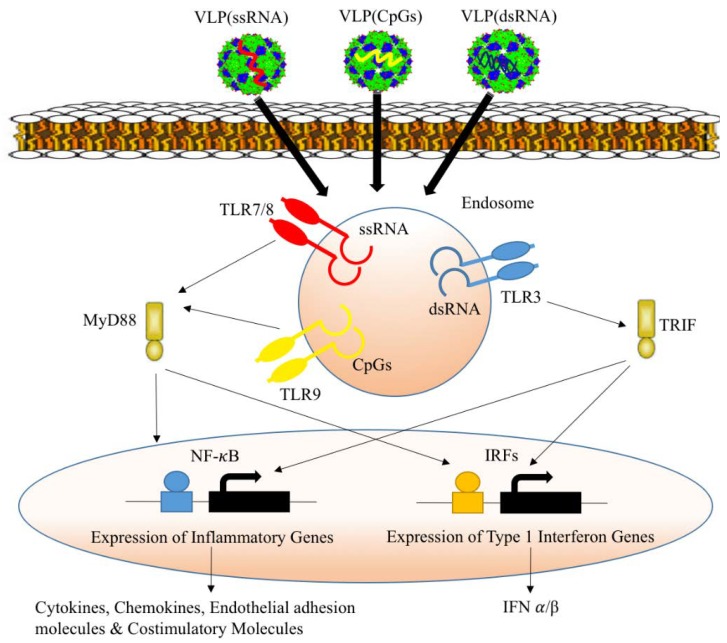
Packaging VLPs with innate immune-modulators. Viral capsid-derived VLPs packaging different nucleic acids can be efficiently internalized by APCs. The packaged nucleic acids will be released in the endosomal compartment of the cell following degradation of VLP-protein shell. ssRNA is TLR7/8 ligand, CpGs is TLR9 ligand and dsRNA is TLR3 ligand. Ligand induced TLR-dimerization activates transcription factors such as NF-κB, AP-1, and IRFs and induces the expression of inflammatory genes and type-I IFN genes.

In general, recognition of ssRNA, dsRNAm or CpGs packaged into VLPs by endosomal TLRs will result in the activation of signaling pathways that are essential for the expression of different genes required to initiate the inflammatory responses (Figure 4). The process starts by ligand-induced TLR dimerization to bring TIR domains in close proximity and facilitate the recruitment of protein kinases. This will activate several major transcription factors such as NF-κB, AP-1, IRF3, and IRF7 [50,51,52,53]. NF-κB and AP-1 will stimulate the expression of several inflammatory genes including cytokines (TNF and IL-6), chemokines (CCL2 and CXCL8), some endothelial adhesion molecules such as E-selectin and other costimulatory molecules including CD80 and CD86. On the other hand, IRF3 and IRF7 are responsible for the expression of type-I IFN α/β genes. TLR7 and TLR9 are MyD88-dependent and IRF-independent signaling pathways, and both are capable of activating NF-κB and IRFs transcription factors. In contrast, TLR3 acts through the activation of TRIF which will induce IRF3 responsible for the expression of type-I IFN [52,53].

Apart from TLRs, other PRRs and nucleic acid sensors have also been reported to play a role in the immune response elicited by VLPs. RIG-I and the stimulator of IFN genes protein (STING) induces significant levels of type-I IFN upon viral infection mediating autocrine and paracrine signaling [54]. Activation of STING results in the activation of different transcription factors including STAT6 and IRF3 [55]. Although STING and RIG-I mediated responses are often associated with intermediates of viral replication, there are evidences that envelope derived VLPs may fuse with cell-membranes which can be sensed by the innate immune cells in murine and human cells. This fusion can activate a STING-dependent signaling pathway which induces the formation of Tank Binding Kinase Complex protein (TBK1) which also involves IRF3. This pathway will finally activate the expression of type-I IFN as well as other IFN stimulating genes (ISGs) [56]. Recently, it has been shown that by including cyclic di-nucleic (CDNs) acids into VLPs, the CTL response against tumors was improved in a cGAS and STING dependent manner in comparison to the effect observed with the addition of the CDNs alone [57]. This highlights the benefits of packaging of nucleic acid into VLPs, which has consistently shown to improve pharmacokinetics of the adjuvants and the observed immune response.

### 2.6. Important Considerations on VLP Based Vaccines

VLPs had been consistently successful at inducing protective immune responses in various pre-clinical models and clinical trials both for infectious and non-infectious diseases. The field had started employing naturally occurring VLPs during infection as vaccines, as exemplified by the Hepatitis B vaccine [58] and has now expanded to well stablished platform VLPs such as the well described Qβ [59], AP205 [41], MS2 [60] to name a few, that had been applied as vaccines against diseases ranging from nicotine dependency [61] to asthma [62] and hypertension [63].

The success of this approach relies on presenting heterologous antigens to the surface of model VLPs and in this manner, conferring the highly immunogenic viral fingerprint to those antigens. There are two main methods for presentation of heterologous antigens: chemical crosslinking or genetic fusion. The choice of method will impact the quality of the immune response as it will affect the valency of decoration of the particle with the antigen and the overall stability of the vaccine. For that reason, small changes in the crosslinking method must be closely evaluated as it can directly affect immunogenicity.

A second important consideration for VLPs is the expression system of VLPs. During the assembly phase VLPs will randomly pack host derived components that can have an unbeknownst impact in the immune response. Thus, the same VLP can exert different immune responses depending on the manufacturing process. This is an important consideration when comparing results across different groups and when scaling up the manufacturing process.

### 2.7. Challenges for VLP-Based Vaccine Development

As is common to other vaccine development platforms, there are several challenges that should be considered for successful development of VLP-based vaccines. There are some risks that are specific to the VLP-platform technologies and some risks that are related to the specific vaccine candidates.

Challenges specific to VLP-based vaccine platforms

- Even though several VLP-based vaccines are on the market, some more recent candidates struggle with stability. In addition, no vaccine that displays foreign epitopes has made it to the market so far. Hence, real-life, market PoC for such vaccines is missing. While there is no a priori reason that this should not be possible, it may still be perceived as a potential risk.
- Most if not all nucleocapsid VLPs derived from RNA viruses package RNA from the production host cells. This may need an additional Quality Control effort.
- If epitopes are to be fused into VLPs, this can create substantial problems, as VLPs may not assemble anymore.

Challenges for individual vaccines

- The selected epitope may not be protective
- Induced immune responses may be too low
- The selected indication may sound interesting but does not attract interest from industry and/or the end-customer

## 3. Conclusions

The highly immunogenic properties of VLPs are a direct consequence of the viral fingerprint retained from the parental viruses. Such properties can be summarized by size, surface geometry, and ability to package nucleic acids. VLPs immunogenicity can also be extended by presenting heterologous antigens on their surface.

## Abbreviations

VLPs	Virus-Like Particles
PRR	Pattern Recognition Receptor
PASP	Pathogen-Associated Structural Pattern
TLR	Toll-Like Receptor
LN	Lymph Node
SCS	Sub-Capsular Sinus
DC	Dendritic Cell
cDC	Conventional Dendritic Cell
pDC	Plasmacytoid Dendritic Cell
dDC	Dermal Dendritic Cell
fDC	Follicular Dendritic Cell
LC	Langerhans Cell
APC	Antigen-Presenting Cell
CTL	Cytotoxic T-Lymphocyte
CRP	C Reactive Protein
PTX3	Pentraxin-Related Protein
MHC-I	Major Histocompatability Class I
MHC-II	Major Histocompatibility Class II
LCMV	Lymphocytic Choriomeningitis Virus
dsRNA	Double Stranded RNA
ssRNA	Single Stranded RNA
TNF	Tumor Necrosis Factor
IFN	Interferon
NF-κB	Nuclear Factor kappa-light-chain-enhancer of Activated B-Cells
AP-1	Activator Protein 1
IRF	Interferon Regulatory factor
CCL2	Chemokine (C-C motif) Ligand 2
CXCL8	Chemokine (C-X-C motif) Ligand 8
MyD88	Myeloid Differentiation Primary Response Gene 88
TRIF	TIR-Domain-Containing Adapter-Inducing Interferon-β

## References

[1] Schwarz B., Uchida M., Douglas T. (2017). Biomedical and Catalytic Opportunities of Virus-Like Particles in Nanotechnology. Adv. Virus Res..

[2] Bachmann M.F., Jennings G.T. (2010). Vaccine delivery: A matter of size, geometry, kinetics and molecular patterns. Nat. Rev. Immunol..

[3] Perlmutter J.D., Hagan M.F. (2015). Mechanisms of virus assembly. Annu. Rev. Phys. Chem..

[4] Goldinger S.M., Dummer R., Baumgaertner P., Mihic-Probst D., Schwarz K., Hammann-Haenni A., Willers J., Geldhof C., Prior J.O., Kundig T.M. (2012). Nano-particle vaccination combined with TLR-7 and -9 ligands triggers memory and effector CD8(+) T-cell responses in melanoma patients. Eur. J. Immunol..

[5] Heo M.B., Kim S.Y., Yun W.S., Lim Y.T. (2015). Sequential delivery of an anticancer drug and combined immunomodulatory nanoparticles for efficient chemoimmunotherapy. Int. J. Nanomed..

[6] Schwarz K., Meijerink E., Speiser D.E., Tissot A.C., Cielens I., Renhof R., Dishlers A., Pumpens P., Bachmann M.F. (2005). Efficient homologous prime-boost strategies for T cell vaccination based on virus-like particles. Eur. J. Immunol..

[7] Brune K.D., Leneghan D.B., Brian I.J., Ishizuka A.S., Bachmann M.F., Draper S.J., Biswas S., Howarth M. (2016). Plug-and-Display: Decoration of Virus-Like Particles via isopeptide bonds for modular immunization. Sci. Rep..

[8] D’Argenio D.A., Wilson C.B. (2010). A decade of vaccines: Integrating immunology and vaccinology for rational vaccine design. Immunity.

[9] Randolph G.J., Angeli V., Swartz M.A. (2005). Dendritic-cell trafficking to lymph nodes through lymphatic vessels. Nat. Rev. Immunol..

[10] Jia R., Guo J.H., Fan M.W. (2012). The effect of antigen size on the immunogenicity of antigen presenting cell targeted DNA vaccine. Int. Immunopharmacol..

[11] Fifis T., Gamvrellis A., Crimeen-Irwin B., Pietersz G.A., Li J., Mottram P.L., McKenzie I.F., Plebanski M. (2004). Size-dependent immunogenicity: Therapeutic and protective properties of nano-vaccines against tumors. J. Immunol..

[12] Reddy S.T., Rehor A., Schmoekel H.G., Hubbell J.A., Swartz M.A. (2006). In vivo targeting of dendritic cells in lymph nodes with poly(propylene sulfide) nanoparticles. J. Control. Release.

[13] Baluk P., Fuxe J., Hashizume H., Romano T., Lashnits E., Butz S., Vestweber D., Corada M., Molendini C., Dejana E. (2007). Functionally specialized junctions between endothelial cells of lymphatic vessels. J. Exp. Med..

[14] Manolova V., Flace A., Bauer M., Schwarz K., Saudan P., Bachmann M.F. (2008). Nanoparticles target distinct dendritic cell populations according to their size. Eur. J. Immunol..

[15] Mohsen M.O., Gomes A.C., Cabral-Miranda G., Krueger C.C., Leoratti F.M., Stein J.V., Bachmann M.F. (2017). Delivering adjuvants and antigens in separate nanoparticles eliminates the need of physical linkage for effective vaccination. J. Control. Release.

[16] Ruedl C., Storni T., Lechner F., Bachi T., Bachmann M.F. (2002). Cross-presentation of virus-like particles by skin-derived CD8(-) dendritic cells: A dispensable role for TAP. Eur. J. Immunol..

[17] Keller S.A., Bauer M., Manolova V., Muntwiler S., Saudan P., Bachmann M.F. (2010). Cutting edge: Limited specialization of dendritic cell subsets for MHC class II-associated presentation of viral particles. J. Immunol..

[18] Qi H., Egen J.G., Huang A.Y., Germain R.N. (2006). Extrafollicular activation of lymph node B cells by antigen-bearing dendritic cells. Science.

[19] Carrasco Y.R., Batista F.D. (2007). B cells acquire particulate antigen in a macrophage-rich area at the boundary between the follicle and the subcapsular sinus of the lymph node. Immunity.

[20] Gonzalez S.F., Degn S.E., Pitcher L.A., Woodruff M., Heesters B.A., Carroll M.C. (2011). Trafficking of B cell antigen in lymph nodes. Annu. Rev. Immunol..

[21] Link A., Zabel F., Schnetzler Y., Titz A., Brombacher F., Bachmann M.F. (2012). Innate immunity mediates follicular transport of particulate but not soluble protein antigen. J. Immunol..

[22] Wykes M., Pombo A., Jenkins C., MacPherson G.G. (1998). Dendritic cells interact directly with naive B lymphocytes to transfer antigen and initiate class switching in a primary T-dependent response. J. Immunol..

[23] Kissenpfennig A., Henri S., Dubois B., Laplace-Builhe C., Perrin P., Romani N., Tripp C.H., Douillard P., Leserman L., Kaiserlian D. (2005). Dynamics and function of Langerhans cells in vivo: Dermal dendritic cells colonize lymph node areas distinct from slower migrating Langerhans cells. Immunity.

[24] Allan R.S., Waithman J., Bedoui S., Jones C.M., Villadangos J.A., Zhan Y., Lew A.M., Shortman K., Heath W.R., Carbone F.R. (2006). Migratory dendritic cells transfer antigen to a lymph node-resident dendritic cell population for efficient CTL priming. Immunity.

[25] Lebel M.E., Langlois M.P., Daudelin J.F., Tarrab E., Savard P., Leclerc D., Lamarre A. (2017). Complement Component 3 Regulates IFN-alpha Production by Plasmacytoid Dendritic Cells following TLR7 Activation by a Plant Virus-like Nanoparticle. J. Immunol..

[26] Andersen O., Vilsgaard Ravn K., Juul Sorensen I., Jonson G., Holm Nielsen E., Svehag S.E. (1997). Serum amyloid P component binds to influenza A virus haemagglutinin and inhibits the virus infection in vitro. Scand. J. Immunol..

[27] Clingan J.M., Matloubian M. (2013). B Cell-intrinsic TLR7 signaling is required for optimal B cell responses during chronic viral infection. J. Immunol..

[28] Jegerlehner A., Maurer P., Bessa J., Hinton H.J., Kopf M., Bachmann M.F. (2007). TLR9 signaling in B cells determines class switch recombination to IgG2a. J. Immunol..

[29] Bessa J., Zabel F., Link A., Jegerlehner A., Hinton H.J., Schmitz N., Bauer M., Kundig T.M., Saudan P., Bachmann M.F. (2012). Low-affinity B cells transport viral particles from the lung to the spleen to initiate antibody responses. Proc. Natl. Acad. Sci. USA.

[30] Bell D., Young J.W., Banchereau J. (1999). Dendritic cells. Adv. Immunol..

[31] Schubert U., Anton L.C., Gibbs J., Norbury C.C., Yewdell J.W., Bennink J.R. (2000). Rapid degradation of a large fraction of newly synthesized proteins by proteasomes. Nature.

[32] Huang A.Y., Bruce A.T., Pardoll D.M., Levitsky H.I. (1996). In vivo cross-priming of MHC class I-restricted antigens requires the TAP transporter. Immunity.

[33] Huang A.Y., Golumbek P., Ahmadzadeh M., Jaffee E., Pardoll D., Levitsky H. (1994). Role of bone marrow-derived cells in presenting MHC class I-restricted tumor antigens. Science.

[34] Storni T., Bachmann M.F. (2004). Loading of MHC class I and II presentation pathways by exogenous antigens: A quantitative in vivo comparison. J. Immunol..

[35] Harding C.V., Song R. (1994). Phagocytic processing of exogenous particulate antigens by macrophages for presentation by class I MHC molecules. J. Immunol..

[36] Fang H., Tan M., Xia M., Wang L., Jiang X. (2013). Norovirus P particle efficiently elicits innate, humoral and cellular immunity. PLoS ONE.

[37] Subklewe M., Paludan C., Tsang M.L., Mahnke K., Steinman R.M., Munz C. (2001). Dendritic cells cross-present latency gene products from Epstein-Barr virus-transformed B cells and expand tumor-reactive CD8(+) killer T cells. J. Exp. Med..

[38] Kovacsovics-Bankowski M., Clark K., Benacerraf B., Rock K.L. (1993). Efficient major histocompatibility complex class I presentation of exogenous antigen upon phagocytosis by macrophages. Proc. Natl. Acad. Sci. USA.

[39] Albert M.L., Sauter B., Bhardwaj N. (1998). Dendritic cells acquire antigen from apoptotic cells and induce class I-restricted CTLs. Nature.

[40] Dudziak D., Kamphorst A.O., Heidkamp G.F., Buchholz V.R., Trumpfheller C., Yamazaki S., Cheong C., Liu K., Lee H.W., Park C.G. (2007). Differential antigen processing by dendritic cell subsets in vivo. Science.

[41] Spohn G., Jennings G.T., Martina B.E., Keller I., Beck M., Pumpens P., Osterhaus A.D., Bachmann M.F. (2010). A VLP-based vaccine targeting domain III of the West Nile virus E protein protects from lethal infection in mice. Virol. J..

[42] Janeway C.A., Medzhitov R. (2002). Innate immune recognition. Annu. Rev. Immunol..

[43] Storni T., Ruedl C., Schwarz K., Schwendener R.A., Renner W.A., Bachmann M.F. (2004). Nonmethylated CG motifs packaged into virus-like particles induce protective cytotoxic T cell responses in the absence of systemic side effects. J. Immunol..

[44] Tabeta K., Georgel P., Janssen E., Du X., Hoebe K., Crozat K., Mudd S., Shamel L., Sovath S., Goode J. (2004). Toll-like receptors 9 and 3 as essential components of innate immune defense against mouse cytomegalovirus infection. Proc. Natl. Acad. Sci. USA.

[45] Akira S., Uematsu S., Takeuchi O. (2006). Pathogen recognition and innate immunity. Cell.

[46] Utaisincharoen P., Kespichayawattana W., Anuntagool N., Chaisuriya P., Pichyangkul S., Krieg A.M., Sirisinha S. (2003). CpG ODN enhances uptake of bacteria by mouse macrophages. Clin. Exp. Immunol..

[47] Hemmi H., Takeuchi O., Kawai T., Kaisho T., Sato S., Sanjo H., Matsumoto M., Hoshino K., Wagner H., Takeda K. (2000). A Toll-like receptor recognizes bacterial DNA. Nature.

[48] Lopez C.B., Moltedo B., Alexopoulou L., Bonifaz L., Flavell R.A., Moran T.M. (2004). TLR-independent induction of dendritic cell maturation and adaptive immunity by negative-strand RNA viruses. J. Immunol..

[49] Schwarz K., Storni T., Manolova V., Didierlaurent A., Sirard J.C., Rothlisberger P., Bachmann M.F. (2003). Role of Toll-like receptors in costimulating cytotoxic T cell responses. Eur. J. Immunol..

[50] Xagorari A., Chlichlia K. (2008). Toll-like receptors and viruses: Induction of innate antiviral immune responses. Open Microbiol. J..

[51] Lester S.N., Li K. (2014). Toll-like receptors in antiviral innate immunity. J. Mol. Biol..

[52] Takeda K., Akira S. (2005). Toll-like receptors in innate immunity. Int. Immunol..

[53] Kawai T., Akira S. (2010). The role of pattern-recognition receptors in innate immunity: Update on Toll-like receptors. Nat. Immunol..

[54] Nakhaei P., Hiscott J., Lin R. (2010). STING-ing the antiviral pathway. J. Mol. Cell Biol..

[55] Burdette D.L., Vance R.E. (2013). STING and the innate immune response to nucleic acids in the cytosol. Nat. Immunol..

[56] Holm C., Jensen S.B., Jakobsen M.R., Cheshenko N., Fitzgerald K.A., Paludan S.R. (2012). Virus-cell fusion as a trigger of innate immunity dependent on the adaptor STING. Nat. Immunol..

[57] Gulen M.F., Koch U., Haag S.M., Schuler F., Apetoh L., Villunger A., Radtke F., Ablasser A. (2017). Signalling strength determines proapoptotic functions of STING. Nat. Commun..

[58] Krugman S. (1982). The newly licensed hepatitis B vaccine. Characteristics and indications for use. JAMA.

[59] Kozlovska T.M., Cielens I., Dreilinna D., Dislers A., Baumanis V., Ose V., Pumpens P. (1993). Recombinant RNA phage Q beta capsid particles synthesized and self-assembled in Escherichia coli. Gene.

[60] Galaway F.A., Stockley P.G. (2013). MS2 viruslike particles: A robust, semisynthetic targeted drug delivery platform. Mol. Pharm..

[61] Maurer P., Jennings G.T., Willers J., Rohner F., Lindman Y., Roubicek K., Renner W.A., Muller P., Bachmann M.F. (2005). A therapeutic vaccine for nicotine dependence: Preclinical efficacy, and Phase I safety and immunogenicity. Eur. J. Immunol..

[62] Akache B., Weeratna R.D., Deora A., Thorn J.M., Champion B., Merson J.R., Davis H.L., McCluskie M.J. (2016). Anti-IgE Qb-VLP Conjugate Vaccine Self-Adjuvants through Activation of TLR7. Vaccines (Basel).

[63] Ambuhl P.M., Tissot A.C., Fulurija A., Maurer P., Nussberger J., Sabat R., Nief V., Schellekens C., Sladko K., Roubicek K. (2007). A vaccine for hypertension based on virus-like particles: Preclinical efficacy and phase I safety and immunogenicity. J. Hypertens..

